# Supplementary material for: Federated learning based reference evapotranspiration estimation for distributed crop fields
Source: PLoS One. 2025 Feb 5;20(2):e0314921. doi: 10.1371/journal.pone.0314921 (PMC11798497; doi:10.1371/journal.pone.0314921)
Supplement: Attachment — Submitted filename: Rebuttal Letter.pdf [file pone.0314921.s002.pdf]

Response to comments and suggestions by reviewers: We sincerely thank the reviewers for providing detailed comments, which have helped us to improve our manuscript's presentation and quality. In the following, we provide a detailed response and changes made for each comment.

As the paper has been revised, it is difficult to append verbatim changes for each response. We have categorically specified the places in the paper which have been changed at the end of each response.

Response to Reviewer 1's Comments:

R1.1: The purpose section should be emphasized better.

Response: Thank you for your valuable suggestion. Accordingly, we have revised the manuscript **(Section 1)**.

R1.2: Give information about the agriculture of the study area.

Response: Thank you for your valuable suggestion. The required information is provided in **Section 3.1 (Page 6, Lines 192-200)**.

R1.3: Write the limitations of the methods.

Response: Thank you for your comment. The limitations of the existing methods are specified in Section 1 (Page 2, Lines 34-37).

R1.4: Add the limitations of the study and your suggestions to the results.

Response: Thank you for the insightful suggestion. Accordingly, we have revised the **Section 4.1 (Page 12, Lines 410-420)** and **Section 5 (Page 13, Lines 464-468)**.

R1.5: It needs a good discussion section separate from results.

Response: Thank you for your comment. Accordingly, the discussion section is separated and revised **(Section 4.1, Page 12, Lines 402-445)**.

R1.6: Test the reliability of prediction results.

Response: Thank you for your comment. To test the reliability of the prediction results, the models were trained and tested using 10-fold cross-validation. We explicitly mentioned it in **Section 3.5 (Page 9, Lines 306-308)**.

R1.7: Compare the measurement and prediction results by performing the Kruskal-Wallis test.

Response: Thank you for your valuable suggestion. Accordingly, Kruskal-Wallis test is performed and added in **Section 4 (Page 11, Lines 355-359)**.

R1.8: Draw the Taylor diagram.

Response: Accordingly, Taylor diagram **(Figure 17)** is added in **Section 4 (Page 11, Lines 370-373)**. Thank you for your comments and suggestions.

R1.9: Draw error boxplot and violin graphs.

Response: Accordingly, error boxplot **(Figure 18 and Figure 19)** and violin graphs **(Figure 6)** are added in **Section 3.2 (Page 11, Lines 374-382)** and **Section 4 (Page 7, Lines 231-240)**, respectively. Thank you for your comments and suggestions.

R1.10: Add the scatter diagrams.

Response: Accordingly, Scatter diagrams (Figures 3-5) are added in **Section 3.2 (Page 7, Line 241-251)**. Thank you for your comments and suggestions.

R1.11: Add descriptive statistics parameters.

Response: Thank you for your valuable suggestion. Accordingly, we have added descriptive statistics parameters in **Section 3.2 (Page 7, Table 2)**.

R1.12: Flowchart is quite pale, needs some improvement.

Response: Thank you for your valuable suggestion. Accordingly, we have improved the Flowchart **Figure 3** (**Figure 14** in the revised submission).

R1.13: Which software did you analyze with?

Response: Thank you for your valuable suggestion. Accordingly, we have added the related details in **Section 4.1 (Page 12, Lines 403-406)**.

R1.14: Can you give information about analysis times? Also provide information about the computer on which you performed the analysis.

Response: Thank you for your valuable suggestion. Accordingly, we have added the related details in **Section 4.1 (Page 12, Lines 403-406)**. .

R1.15: Include descriptive statistics of the data.

Response: Thank you for your valuable suggestion. Accordingly, we have added descriptive statistics parameters in **Section 3.2 (Page 7, Table 2)**.

R1.16: Add NSE to the Evaluation criteria section. Interpret the MAPE, R2 and NSE criteria according:
Uncuoglu, E., Citakoglu, H., Latifoglu, L., Bayram, S., Laman, M., Ilkentapar, M., & Oner, A. A. (2022). Comparison of neural network, Gaussian regression, support vector machine, long short-term memory, multi-gene genetic programming, and M5 Trees methods for solving civil engineering problems. Applied Soft Computing, 129, 109623.

Response: Thank you for your valuable suggestion. Accordingly, we have added NSE to the evaluation metrics **(Section 3.5, Page 10, Lines 331-335)** and revised the results accordingly **(Table 4, Page 12) and (Section 4 and 5, Page 10-12)**.

R1.17: Check out the following articles about
Bayram, S., & Çıtakoğlu, H. (2023). Modeling monthly reference evapotranspiration process in Turkey: application of machine learning methods. Environmental Monitoring and Assessment, 195(1), 67.
Citakoglu, H., Cobaner, M., Haktanir, T., & Kisi, O. (2014). Estimation of monthly mean reference evapotranspiration in Turkey. Water Resources Management, 28, 99-113.
Cobaner, M., Citakoğlu, H., Haktanir, T., & Kisi, O. (2017). Modifying Hargreaves–Samani equation with meteorological variables for estimation of reference evapotranspiration in Turkey. Hydrology Research, 48(2), 480-497.

Response: Thank you for your valuable suggestion. Accordingly, we have revised the manuscript **(Section 1, Page 2, Lines 19-23)**.

Response to Reviewer 2's Comments:

R2.1: The introduction lacks recent literature updates. It is essential to incorporate recent studies to provide a comprehensive overview of the current state of research in the field. Below are suggested paragraphs to be added to the literature review section to enhance the introduction: "Machine learning Remote sensing field becomes cost effective tools in modeling hydrological variables e.g. River discharge, rainfall runoff estimation, Evapotranspiration, land use classification, surface temperature estimation". Following studies will enrich the introduction and literature sections:

1. https://doi.org/10.3390/rs12071107
2. https://doi.org/10.1029/2023WR035785
3. https://link.springer.com/article/10.1007/s43621-024-00263-w
4. https://doi.org/10.1201/9781003377825 (Chapter 3)
5. https://doi.org/10.1007/s42452-023-05520-7
6. https://doi.org/10.1186/s40562-023-00287-6
7. https://doi.org/10.3390/su15043572
8. https://doi.org/10.1007/978-3-031-29394-8_8
9. https://doi.org/10.3390/atmos13101609

Response: Thank you for your valuable Suggestion. Accordingly, we have added recent studies to improve the Introduction **(Section 1, Page 2, Line 19-23)** and Literature Review **(Section 2, Page 5, Lines 170-176)**.

R2.2: Novelty of the paper is not written clearly. It should be highlighted.

Response: Thank you for your comment. The novelty of paper is clearly specified in **Section 1 (Page 3, lines 59-75)**.

R2.3: How you divide the dataset? How much data is used for training and testing periods? I suggest authors to add table along with statistics in dataset sections.

Response: Thank you for your valuable suggestion. We have specified the distribution of the training and testing dataset in **Section 3.5 (Page 9, Lines 306-308)** and descriptive statistics parameters are provided in **Section 3.2 (Page 7, Table 2)**.

R2.4: I did not find adequate information on the methodologies related to Random Forest Regressor (RFR), Support Vector Regressor (SVR), and Decision Tree Regressor (DTR). Although I found some information on the Federated Learning (FL) model, it is not sufficient for the focus of this paper, which centers around Federated Learning.

Response: Thank you for your comment. Accordingly, we have added the information on the methodology in **Section 3.3 (Page 8, Lines 257-278)**.

R2.5: Compare your findings with previous stud and visual representation (Taylor diagram, Radar chart, Smith graph) of machine learning results should be added. Below are the reference studies and need to be citied:

1. https://doi.org/10.1007/s13201-022-01667-7
2. https://www.mdpi.com/2073-4441/14/10/1666
3. https://www.mdpi.com/2306-5338/10/8/169

4. https://www.mdpi.com/2073-4441/15/6/1149
5. https://doi.org/10.1002/ird.2838

Response: Thank you for your comments. Accordingly, we have added the suggested visual representation and its detail in **(Taylor Diagram (Figure 17), Section 4, Page 11, Lines 370-373)** and **(Radar Chart (Figures 15-16) Section 4, Page 11, Lines 361-369)**. we also have specified the reason for avoiding the Smith Graph in **Section 4, Page 11, Lines 367-369)**.

R2.6: Some grammatical errors are present and need revision.

Response: Thank you for your comments. Accordingly, we have carefully read the manuscript to mitigate the grammatical errors and typos using Grammarly.

R2.7: Clear location map in Figure 2 is needed.

Response: Thank you for your comments. The location map (**Figure 2**) is revised in Python (No copyright is required).

R2.8: Improvement in the graphical representation of Figure 3.

Response: Thank you for your comments. **Figure 3** (**Figure 14** in the revised manuscript) is improved accordingly.

R2.9: Check the references as per journal guidelines and add DOI in references.

Response: Thank you for your comments. Accordingly, PLOS One Journal guidelines are checked, and **DOIs** are added to the references.
