# Supplementary material for: Federated learning based reference evapotranspiration estimation for distributed crop fields
Source: PLoS One. 2025 Feb 5;20(2):e0314921. doi: 10.1371/journal.pone.0314921 (PMC11798497; doi:10.1371/journal.pone.0314921)
Supplement: Attachment — Submitted filename: Response to Reviewers.pdf [file pone.0314921.s004.pdf]

Response to comments and suggestions by reviewers: We sincerely thank the reviewers for providing detailed comments, which have helped us to improve our manuscript's presentation and quality. In the following, we provide a detailed response and changes made for each comment.

As the paper has been revised, it is difficult to append verbatim changes for each response. We have categorically specified the places in the paper which have been changed at the end of each response.

Response to Editor's Comments:

R2.1: Abbreviations in Table 1: Many abbreviations are used and not included in the Abbreviations List.

Response: thank you for your comment. Accordingly, the missing abbreviations are added in the **Section "Abbreviations and Acronyms" (Page 15, Lines 523-542)**.

R2.2: Line 211: Specify which observed data were collected from the selected stations and which equation was used to calculate the daily reference ETo.

Response: We apologize for neglecting the issue. Accordingly, we have explicitly specified the used equation in **Section 3.2 (Page 6, Line2 213-214)**.

R2.3: Equations 5 to 8: The index "I" or "t" is missing.

Response: Thank you for your valuable comment. We have revised the manuscript accordingly **(Section 3.5, Pages 10-11, Lines 346-47, 352-53, 362)**.

R2.4: Figure 17: The Taylor diagram is incorrectly presented; the x and y axes should be the standard deviation (STD), and the radial axis should represent the correlation (R). **Response:** Thank you for your comment. Our Taylor diagram accurately follows standard conventions, with the radial axis representing standard deviation (STD) and the angular coordinate showing correlation (R). This structure is consistent with established practices, as detailed in Kenneth E. Taylor's "Summarizing Multiple Aspects of Model Performance in a Single Diagram" (2001). If specific aspects of our diagram seem unclear, we are open to further clarification or adjustments as needed.

R2.5: Figure 21: This figure does not provide additional information. The results in Table 4 are more informative.

Response: Thank you for your valuable comment. Accordingly, we removed the manuscript's **Figure 21**.

R2.6: Sentences (from line 54 to 57 and from 284 to 286) need references

Response: The references [17, 63] are added. Thank you for your comment.

Response to Reviewer 3 Comments:

R3.1: Time spent need to be measured in the experimental results.

Response: Thank you for your comment. Accordingly, we have explicitly mentioned the time spent in **Section 4.1 (Page 12, Lines 432-437)**.

R3.2: Limitation and Discussion Sections need to be inserted.

Response: Thank you for your valuable suggestion. Accordingly, we have added the Limitations in the **Last Paragraph of Section 4.1 (Page 14, Lines 480-488)**.

R3.3: All metrics need to be calculated in the experimental results.

Response: Thank you for your comment. All the metrics are calculated and reported in the experimental results **(Table 4 (Page 13))** and discussed in the **Last Paragraph of Section 4 (Page 12, Lines 418-430)**.

R3.4: The parameters used for the analysis must be provided in table.

Response: Thank you for your comment. The parameters used for analysis and their statistic analysis are provided in **Table 2 (Page 7)**.

R3.5: Comparison with similar studies on a similar dataset need to be inserted (with references).

Response: Thank you for your comment. Notably, to our knowledge, the proposed FL method is the first to automatically estimate ET_o for distributed fields using Federated Learning (FL). Therefore, it is compared with traditional machine learning models instead of baseline models.

We have explicitly specified it in Section 4 **(Pages 11, Lines 368-371)**.

R3.6: The cost associated with deploying these deep learning models, including the necessary hardware and software, is not addressed.

Response: Thank you for your comment. Accordingly, we have addressed the cost of deploying these deep learning models, including the necessary hardware and software in **Section 4.1 (Page 12, Line 432-437)**.

R3.7: The architecture of the proposed model must be provided.

Response: Thank you for your valuable suggestion. Although the architecture is illustrated in **Figure 1** and **Figure 14**, and its working is presented in **Algorithm 1**, we have explicitly added and explained the architecture of the proposed model in the explanation of **Algorithm 1 (Page 9-10, Lines 316-329)**.

R3.8: The authors need to make a clear proofread to avoid grammatical mistakes and typo errors.

Response: Thank you for your comment. Accordingly, we have proofread to avoid grammatical and typo mistakes.

R3.9: Enhance the clarity of the Figures by improving their resolution.

Response: Fixed. Thank you for your comment.

R3.10: Add future work in last section (conclusion) (if any).

Response: Thank you for your comment. The manuscript is revised accordingly **(Section 5, Page 14, Lines 500-502)**.

R3.11: To improve the Related Work and Introduction sections authors are recommended to review this highly related research work paper.
a) Feature reduction for hepatocellular carcinoma prediction using machine learning algorithms
b) Detecting cyberbullying using deep learning techniques: a pre-trained glove and focal loss technique
c) Optimal Gasoline Price Predictions: Leveraging the ANFIS Regression Model
d) The effect of rebalancing techniques on the classification performance in cyberbullying datasets
e) Developing an efficient method for automatic threshold detection based on hybrid feature selection approach
f) Acoustic data detection in large-scale emergency vehicle sirens and road noise dataset
g) Employing machine learning for enhanced abdominal fat prediction in cavitation post-treatment
h) Predicting female pelvic tilt and lumbar angle using machine learning in case of urinary incontinence and sexual dysfunction

Response: Thank you for your suggestions to improve the work. Due to editor instruction, we are unable to incorporate changes. Editor has restricted in this manner. Hope you will understand.

Response to Reviewer 4 Comments:

R4.1: I do not understand why you recommend federated learning over centralized learning for estimating reference evapotranspiration. The title is "Federated learning based reference evapotranspiration estimation for distributed crop fields". It means that you are proposing a federated learning, which is suitable for heterogeneous datasets whose sizes may span several orders of magnitude, for estimating a variable in three location (distributed) with quite similar datasets. What is wrong with applying distributed learning for estimating reference evapotranspiration.

Response: Thank your for your comment. By training local models and sharing their insights, federated learning helps create more accurate ETo estimates that work well in different locations. Even if the datasets look similar, small differences in local conditions, soil types, or weather patterns can impact ETo estimates. The detailed reasons for choosing federated learning over machine/deep learning are mentioned in **Section 1 (Page 2, Lines 49-55)**.

R4.1: The abstract does not justify why there is a need to apply a federated learning for estimating reference evapotranspiration.

Response: Thank you for your comment. We have revised the **Abstract** accordingly.

R4.2: The gap in the literature is not established in the introduction.

Response: Thank you for your comment. We have established the gap in the literature in **Introduction (Section 1, Page 2, Lines 35-49)**. .

R4.3: Remove Equation 1 from the introduction and present it in the materials and methods section.

Response: Equation 1 is moved from the **Introduction (Section 1)** to **materials and methods (Section 3, Equation 1, Lines 184-185)**.

R4.4: It is stated in lines 35-37 that "Moreover, simple ML approaches also face limitations, particularly related to data centralization and may not effectively capture the diverse climatic conditions of different regions." For this case, why do not we add climatic conditions as an input instead of considering a federated learning for estimating reference evapotranspiration.

Response: We apologize for neglecting the issue. Accordingly, we have explicitly specified the reason why we do not add climatic conditions as an input instead of considering federated learning for estimating reference evapotranspiration in **Section 3.2 (Page 7, Lines 220-222)**.

R4.5: I suggest merging the section of "2 Related work" within the introduction.

Response: Thank you for your suggestion. Notable, we have separated the Introduction and Related Work sections as per the requirements of the PLoS One journal.

R4.6: 177-184: This paragraph is repeating the last paragraph of the introduction. Avoid repeating. Methods:

Response: Thank you for your valuable suggestion. The reputation is removed as suggested **(Section 2, Page 5, Lines 177-179)**.

R4.7: Lines 285-286: ". . . . which has proven to be a viable solution for overcoming the challenges mentioned earlier and improving ETo estimates." This sentence requires a reference. Otherwise, you have to justify it.

Response: The reference [**63**] is added. Thank you for your comment.

R4.8: There are a lot figures in this section, while very little description is provided in the text. Explain what every figure/table shows in details in the text.

Response: Thank you for your valuable suggestion. We have revised the manuscript accordingly **(Section 3.2, Page 6-8, Lines (217-219, 234-238, 247-248, 258-262))**.

R4.9: Figure 14 is not a good flowchart for the FL. It does not show how you integrate the local models.

Response: Thank you for your comment. Accordingly, we have revised **Figure 14**.

R4.10: Line 301: Remove " from the end of the sentence.

Response: **Fixed**. Thank you for your comment.

R4.11: Check Equation 3 and introduce all parameters in this equation appropriately.

Response: Thank you for your comment. Accordingly, the parameters of equation 3 are defined in **Section 3.4 (Page 9, Lines 313-315)**.

R4.12: Lines 303-304: Where is the description of this part?.

Response: Thank you for your valuable comment. Accordingly, we have added the description of **Algorithm 1 (Pages 9-10, Lines 316-329)**.

R4.13: Add an appropriate reference for any equation that it is not from your work

Response: Thank you for your comment. Accordingly, we have double-checked the equations used from the literature and cited them wherever required.

R4.14: More information should be added regarding how you calibrate the hyperparameters of the ML models.

Response: Thank you for your comment. Accordingly, the suggested information is added in **Section 3.4 (Pages 9-10, Lines 316-319)**.

R4.15: Which software/libraries you used to implement the ML algorithms and FL?.

Response: Thank you for your comment. We have explicitly specified the libraries used to implement ML/FL algorithms in Section 4.1 (Page 12, Lines 432-433),

R4.16: Add a reliability analysis (10.1007/s40899-023-01021-y) to check how reliable the estimations made by your ML models are.

Response: Thank you for your comment. The analysis presented, while thorough and insightful, is not well-suited for implementation within a federated learning framework. As a result, this approach was not included in the study. However, we acknowledge the value of exploring alternative methods and remain open to considering other techniques in future research.

R4.17: How much did FL model improve estimated reference evapotranspiration in comparison to using individual ML models?.

Response: Thank you for your comment. Accordingly, we have revised the manuscript **(Section 4.1, Pages 13-14, Lines 476-479)**.

R4.18: There are a lot figures in this section, while very little description is provided in the text. Explain what every figure/table shows in details in the text

Response: Thank you for your valuable suggestion. We have revised the manuscript accordingly **(Section 3.2, Page 6-8, Lines (217-219, 234-238, 247-248, 258-262))**.

R4.19: What are the advantages and disadvantages of the FL model?

Response: Thank you for your comment. The manuscript is revised accordingly (Section 1, Page 2-3, 56-64).

R4.20: What is the practical contribution of your work? How does it contribute to the practical field in the realm of improving water balance analysis.

Response: Thank you for your comment. The manuscript is revised accordingly (Section 1, Page 3, 64-67).

R4.21: The discussion should merge the findings of this work with other ones available in the literature.

Response: Thank you for your comment. Notably, to our knowledge, the proposed FL method is the first to automatically estimate ET_o for distributed fields using Federated Learning (FL). Therefore, it is compared with traditional machine learning models instead of models available in the literature.

R4.22: The conclusion should contain general findings from the study and present some take-home messages. Improve this section.

Response: Thank you for your valuable suggestion. The **conclusion section (Page 14, Lines 490-502)** is revised accordingly.
