# Supplementary material for: Federated learning based reference evapotranspiration estimation for distributed crop fields
Source: PLoS One. 2025 Feb 5;20(2):e0314921. doi: 10.1371/journal.pone.0314921 (PMC11798497; doi:10.1371/journal.pone.0314921)
Supplement: Attachment — Submitted filename: Response to Reviewer.pdf [file pone.0314921.s005.pdf]

Dear Reviewers and Editor

I hope this message finds you well. We are pleased to submit the revised version of our manuscript. Thank you for your insightful feedback on our manuscript. We appreciate the time and effort you dedicated to your reviews. We sincerely appreciate the valuable feedback provided by you and the reviewers. We have carefully addressed all comments and suggestions, which we believe have significantly improved the manuscript. Below, we outline the key changes made in response to the reviewers' feedback:

Reviewer #1:

We are grateful for your positive comments regarding “all comments are addressed”and for recognizing our efforts in addressing previous concerns. Your support is invaluable.

Reviewer #4:

Thank you for your thorough review. We appreciate your points on federated learning (FL). We have clarified our justification for FL over traditional methods, emphasizing its advantages in privacy preservation and collaborative training without sharing raw data. We have expanded this discussion in the revised manuscript.

Comment 1:

The authors' responses are not detailed and some of the major comments were not addressed properly. For instance, they state in lines 41-42: "A universal ETo model cannot be created using traditional machine learning methods because they can’t handle diverse weather conditions."This is not correct. There are many papers on application of traditional machine learning methods for this purpose. It is not the gap in the literature. In addition, the authors' response to the comment why they applied "federated learning over centralized learning for estimating reference evapotranspiration" is not acceptable. It is important to note that there are many machine learning models and artificial intelligence techniques. Nevertheless, they should be used for solving appropriate problems. In other words, the authors proposed federated learning for estimating reference evapotranspiration, while it is not justified. Instead of federated learning, distributed learning, which is a suitable tool. can be used. Since the major contribution of this manuscript is proposinjg federated learning for estimating reference evapotranspiration, I suggest rejection.

Response:

Agreed; the contribution and justification of FL model is updated with highlighted changes on page 2 in introduction section, and on page page 5 and 6 keeping in view your concern. Hope the clear emphasis clear contribution and suggest use of FL model.

The discussion section is also updated to reflect the reasons fo not choosing the distributed approach on page 16.

Reviewer #5:
The response to your comments and suggestions are given below;

Comment 1:
The author mentions that a "feature importance-based analysis" was conducted; however, the manuscript lacks details on how the importance of the features was calculated. Please provide a clear explanation and justification for the method used to determine feature importance, including any metrics, algorithms, or criteria employed in the analysis.
Response:
Agreed; the description regarding the method used to calculate the Feature importance is given on page 9 and Page 10 with highlighted changes.

Comment 2:
The manuscript lacks technical details about the proposed Federated Learning-based framework for estimating ETo. Readers would benefit from more specific information about the framework's design and implementation. For example, what type of architectural design was used on the client side in FL environment for estimating ETo? How many clients participated in the training process? How many communication rounds occurred between the server and clients to exchange trained weights? Additionally, if machine learning algorithms such as Random Forest Regressor (RFR), Decision Tree Regressor (DTR), or Support Vector Regressor (SVR) were trained on the client side, it is important to explain how the trained weights were aggregated on the server side, evaluated, and then sent back to the clients. Please provide appropriate details to address these concerns, and if possible, include code to further clarify your approach.
Response:
Agreed the details of federated learning model is added on page 10,11 with highlighted changes.

We believe these revisions address your concerns and enhance the clarity of our work. Thank you once again for your valuable contributions. We look forward to your feedback on the revised manuscript.

Best regards,
Muhammad Tausif et al.
