# Supplementary material for: Federated learning based reference evapotranspiration estimation for distributed crop fields
Source: PLoS One. 2025 Feb 5;20(2):e0314921. doi: 10.1371/journal.pone.0314921 (PMC11798497; doi:10.1371/journal.pone.0314921)
Supplement: Attachment — Submitted filename: Response to Reviewers.pdf [file pone.0314921.s006.pdf]

Response to comments and suggestions by reviewers: We sincerely thank the reviewers for providing detailed comments, which have helped us to improve our manuscript's presentation and quality. In the following, we provide a detailed response and changes made for each comment.

As the paper has been revised, it is difficult to append verbatim changes for each response. We have categorically specified the places in the paper which have been changed at the end of each response.

Response to Reviewer 1's Comments:

R1.1: The authors of the article have satisfactorily incorporated all my comments and I have no further comments.

Response: Thank you for your time and efforts to improve the manuscript.

Response to Reviewer 4's Comments:

R4.1: The justification of using federated learning seems to be improved. But not satisfactory because the results of traditional ML models rely on their input data. I recommend to modify this sentence: "Traditional machine learning methods of ETo are very effective in many contexts but limited in the scope of their applicability to specific geographical locations". When you provide traditional ML models with a local dataset, they cannot be used globally. Therefore, this is a problem related to the input data rather than ML models. Additionally, there is no postprocessing of the ML results. Their preformances were copared only based on a few metrics. Therefore, there is a need to conduct futher analyses on the ML results, like reliability analysis or uncertainty analysis (10.1007/s40899-023-01021-y and 10.1016/j.compag.2020.105653). This is neccessary to do because you are comparing different types of ML models. Also, this can improve the discussion section and provide a better perspective of the results.

Response: Thank you for you comments and suggestion.

We have revised the mentioned statement as per your suggestion **(Page 2, Lines 47-51)**.

Moreover, we perform **Kruskal-Wallis** to evaluate the performance of RFR, SVR and DTR and **ANOVA** test to analyze the reliability of the proposed approach. The results of ANOVA analysis are presented on **Page 14 (Lines 488-494)**.

Response to Reviewer 5's Comments:

R5.1: The authors' responses to the review comments lack sufficient detail, and my major points raised in the previous review have not been adequately addressed. There is a significant absence of technical details throughout the manuscript. Although the authors claim to utilize Federated Learning approaches for estimating evapotranspiration in distributed crop fields, they fail to provide specific information on how this technology is implemented within their study. The manuscript does not explain in detail how the methodology used and the steps taken to integrate Federated Learning into their analysis. Furthermore, the responses provided mainly discuss the general principles of Federated Learning rather than its application to the authors' specific research context. This generic explanation does not demonstrate how the authors' approach is uniquely tailored to their work, making it challenging to assess

the validity and relevance of their findings. To enhance the manuscript, I recommend that the authors include technical descriptions of their methodology, particularly, how Federated Learning was specifically applied to their evapotranspiration estimation. This information is crucial for readers to understand and evaluate the effectiveness of the proposed approach.

Response: Thank you for your valuable suggestion. Accordingly, we have revised the Section 3.5 (Pages 10-11, Lines 353-416).
