# Supplementary material for: Federated learning based reference evapotranspiration estimation for distributed crop fields
Source: PLoS One. 2025 Feb 5;20(2):e0314921. doi: 10.1371/journal.pone.0314921 (PMC11798497; doi:10.1371/journal.pone.0314921)
Supplement: Attachment — Submitted filename: Review of the Manuscript PONE-D-24-15636R1.docx [file pone.0314921.s003.docx]

**Review of the Manuscript PONE-D-24-15636R1: "Federated Learning-Based Reference Evapotranspiration Estimation for Distributed Crop Fields"**

**Content Remarks**

The manuscript titled "Federated Learning-Based Reference Evapotranspiration Estimation for Distributed Crop Fields" addresses an important and timely problem in the field of irrigation water management. The authors propose a novel approach using federated learning to estimate reference evapotranspiration (ETo) across three geographical locations with diverse weather conditions. This method aims to improve the accuracy and generalizability of ETo predictions, which are critical for efficient water resource management and sustainable agriculture.

**The introduction**

The introduction effectively sets the stage for the study by highlighting the importance of accurate ETo estimation for agriculture and water management. The authors also state the limitations of existing machine learning approaches that are typically confined to specific regions. The introduction could be further strengthened by providing a brief literature overview of federated learning and its advantages over traditional centralized models in the context of this study.

**The Methodology**

The methodology section is comprehensive. The authors selected three distinct geographical locations in Pakistan; Multan, Faisalabad, and Rawalpindi to implement their federated learning model using weather data from 2012 to 2022. They evaluated three machine learning models: Random Forest Regressor (RFR), Support Vector Regressor (SVR), and Decision Tree Regressor (DTR) against the corresponding federated learning (FL) models. The description of the federated learning process, including the initialization of global model parameters and the iterative updating of local models, is clear and methodical.

**The Data and Results**

The authors provided descriptive statistics for the datasets used from the three locations, including variables such as maximum temperature (Tmax), wind speed (WS), relative humidity (RH), and ETo. The statistical summary indicates significant variability across regions, which underscores the challenge of creating a unified model. The federated learning approach, despite this variability, aims to produce a model that generalizes well across different regions.

The results are clearly presented, showing the performance metrics (R2, RMSE, MAE, MAPE, NSE) for each model and dataset. The Random Forest Regressor (RFR) consistently outperformed the other models both locally and in the federated approach. However, the federated model's RMSE increased, indicating potential areas for improvement in the aggregation method.

Contrary to what the authors mentioned, the results reveal that the federated learning model's performance is lower than individual local models, particularly in terms of RMSE and slightly in terms of R2.

-R² Decrease: The decreases in R² for Faisalabad (2%), Multan (3%), and Rawalpindi (1%) are within a generally acceptable range for many applications, especially if the benefits of federated learning are significant.

- RMSE Increase: The RMSE increases, particularly for Faisalabad (58%) and Rawalpindi (32%), are substantial. This suggests that while the R² decrease might be acceptable, the increases in RMSE are more problematic and indicate a significant reduction in model accuracy.

This performance drop could be due to the simple averaging of weights, which does not account for the heterogeneity of the data. It does not consider the variability and importance of different clients' data, leading to suboptimal model performance.

**The Discussion**

The discussion section effectively interprets the results, acknowledging the limitations of traditional ML methods but not adequately addressing those of the federated learning approach used in the study. There is a performance drop in the federated model, possibly due to the simple averaging of weights. The manuscript would benefit from a more in-depth explanation of why federated learning based on simple averaging of weights may lead to suboptimal performance. The authors should discuss potential solutions or alternative aggregation methods to address this issue. The discussion could be enhanced by including a comparison with other studies that have used federated learning in similar contexts.

**The Conclusion**

The conclusion summarizes all the findings and reiterates the potential of federated learning for ETo estimation across diverse regions.

**Recommendations**

1. Detailed Explanation of Federated Learning Aggregation Methods: Include a more thorough explanation of the potential drawbacks of simple weight averaging and discuss alternative aggregation methods.

2. Comparison with Existing Studies: Provide a comparison with other studies that have applied federated learning in similar fields to contextualize the results.

3. Additional Insights on Data Variability**: Discuss how the variability in the data from different regions specifically impacts the model performance and suggest ways to mitigate these effects.

**Overall Evaluation**

The manuscript explores a significant problem in agricultural water management using a novel approach. While the federated learning model shows promise, the performance drop highlights the need for more sophisticated methods. With the recommended enhancements, the manuscript would make a valuable contribution to the field.

**Final Remarks**

The authors have made a commendable effort in exploring federated learning for ETo estimation. This study opens up new avenues for research and applications in sustainable agriculture and water resource management. By addressing the identified limitations and incorporating the suggested improvements, the manuscript will provide even greater insights and utility to researchers and practitioners in the field.

**Editing Remarks**

1. Abbreviations in Table 1: Many abbreviations are used and not included in the Abbreviations List.

2. Line 211: Specify which observed data were collected from the selected stations and which equation was used to calculate the daily reference ETo.

3. Equations 5 to 8: The index "I" or "t" is missing.

4. Figure 17: The Taylor diagram is incorrectly presented; the x and y axes should be the standard deviation (STD), and the radial axis should represent the correlation (R).

5. Figure 21: This figure does not provide additional information. The results in Table 4 are more informative.

6. Sentences (from line 54 to 57 and from 284 to 286 ) need references
