# Supplementary material for: Federated learning based reference evapotranspiration estimation for distributed crop fields
Source: PLoS One. 2025 Feb 5;20(2):e0314921. doi: 10.1371/journal.pone.0314921 (PMC11798497; doi:10.1371/journal.pone.0314921)
Supplement: Attachment — Submitted filename: Reviewer Comments -1.docx [file pone.0314921.s001.docx]

In the present paper, the authors propose an interesting approach for reference evapotranspiration estimation for distributed crop fields using Federated learning based techniques. The paper seem to be interesting and needs to be improved before publication. Following are the major and minor and improvements:

**Major comments**

I. The introduction lacks recent literature updates. It is essential to incorporate recent studies to provide a comprehensive overview of the current state of research in the field. Below are suggested paragraphs to be added to the literature review section to enhance the introduction:

"Machine learning Remote sensing field becomes cost effective tools in modeling hydrological variables e.g. River discharge, rainfall runoff estimation, Evapotranspiration, land use classification, surface temperature estimation". Following studies will enrich the introduction and literature sections:

1. <https://doi.org/10.3390/rs12071107>
2. <https://doi.org/10.1029/2023WR035785>
3. <https://link.springer.com/article/10.1007/s43621-024-00263-w>
4. <https://doi.org/10.1201/9781003377825> (Chapter 3)
5. <https://doi.org/10.1007/s42452-023-05520-7>
6. <https://doi.org/10.1186/s40562-023-00287-6>
7. <https://doi.org/10.3390/su15043572>
8. <https://doi.org/10.1007/978-3-031-29394-8_8>
9. <https://doi.org/10.3390/atmos13101609>
II. Novelty of the paper is not written clearly. It should be highlighted.
III. How you divide the dataset? How much data is used for training and testing periods? I suggest authors to add table along with statistics in dataset sections.
IV. I did not find adequate information on the methodologies related to Random Forest Regressor (RFR), Support Vector Regressor (SVR), and Decision Tree Regressor (DTR). Although I found some information on the Federated Learning (FL) model, it is not sufficient for the focus of this paper, which centers around Federated Learning.
V. Compare your findings with previous stud and visual representation (Taylor diagram, Radar chart, Smith graph) of machine learning results should be added. Below are the reference studies and need to be citied:
1. <https://doi.org/10.1007/s13201-022-01667-7>
2. <https://www.mdpi.com/2073-4441/14/10/1666>
3. <https://www.mdpi.com/2306-5338/10/8/169>
4. <https://www.mdpi.com/2073-4441/15/6/1149>
5. <https://doi.org/10.1002/ird.2838>

**Minor comments**

Some grammatical errors are present and need revision.

Clear location map in Figure 2 is needed.

Improvement in the graphical representation of Figure 3.

Check the references as per journal guidelines and add DOI in references
